# Growth and Generation Inclusion: From Generation X Nurse Managers' Leadership Experience for Generation Z Nurses

**DOI:** 10.1155/jonm/8430633

**Published:** 2025-10-04

**Authors:** Kuo-Min Chu, Shiow-Ching Shun, Chin-Ying Huang, Li-Hwa Lin, Hsiu-Chu Hsu

**Affiliations:** ^1^Department of Nursing, Taipei Veterans General Hospital, Taipei, Taiwan; ^2^School of Nursing, College of Medicine, National Taiwan University, Taipei, Taiwan; ^3^Institute of Clinical Nursing, National Yang Ming Chiao Tung University, Taipei, Taiwan; ^4^Deputy Superintendent of the Hospital, Taipei Municipal Gan-Dau Hospital, Taipei, Taiwan

**Keywords:** generation X nurse manager, generation Z nurses, inductive thematic analysis, leadership experience

## Abstract

**Background:**

Generation X nurse managers (NMs) face challenges arising from the unique work values and needs presented by Generation Z nurses, who are the newest members of the nursing workforce. However, research on how they adapt to and lead Generation Z nurses remains limited.

**Aim:**

This study aimed to explore and gain an in-depth understanding of the experiences and growth of Generation X NMs in Taiwan as they lead Generation Z nurses.

**Methods:**

This study employed a qualitative approach, using purposive sampling to recruit participants and conducting in-depth semistructured interviews with 11 NMs. All interviews were audiorecorded with participant consent, transcribed verbatim, and analyzed using an inductive thematic approach.

**Results:**

The Generation X NMs described the challenges and growth they experienced while leading Generation Z nurses, which included four main themes: comparative experiences, dialectics of value, sense of mission and social responsibility, and support system.

**Conclusion:**

This study highlights the complex and meaningful journey undertaken by Generation X NMs in Taiwan as they lead Generation Z nurses. Despite notable differences in work attitudes and values between the two generations, these NMs actively promote generational reconciliation and integration. Motivated by a strong sense of mission and social responsibility, their efforts focus on improving the retention rate of Generation Z nurses. The NMs' initial intentions, along with the support they receive from their superiors, serve as key enablers in facilitating this process.

**Implications for Nursing Management:**

Healthcare organizations and policymakers should consider the experiences and needs of NMs while formulating nurse retention policies. By providing essential training programs and emotional support to enhance their management skills, they can effectively promote the retention and development of Generation Z nurses.

## 1. Introduction

The nursing workforce today spans multiple generations, including Baby Boomers (BB, born 1945–1964), Generation X (born 1965–1980), Generation Y (born 1981–1996), and Generation Z (born after 1996) [1]. As BB nurses retire, nurses from Generations X and Y are increasingly assuming the role of nurse managers (NMs), taking on the responsibility of leading the new generation of nurses [2–4]. Generation Z nurses, the youngest cohort in today's nursing workforce, have distinct characteristics, expectations [5], and technology-oriented traits that present new challenges for NMs and human resource planning [6]. In healthcare systems, NMs serve as a bridge between hospital administration and nursing staff. They are responsible for guiding and supervising clinical care [2, 7]; motivating, coaching, and leading staff to achieve organizational goals [8, 9]; improving patient care outcomes; and enhancing nurse satisfaction and retention [1, 8]. Therefore, understanding NMs' experiences is vital for addressing generational differences, fostering effective leadership, and ensuring a supportive work environment that meets the evolving needs of the nursing workforce. Owing to the mandatory retirement age of 65 years in Taiwan's public hospitals, most current clinical NMs are from Generation X. Therefore, they are well-positioned to offer representative and valuable perspectives and experiences in leading Generation Z nurses.

Generation X now predominantly occupies managerial positions and plays a central role in organizational decision-making [10]. As “digital immigrants,” they have experienced the transformation of information and technology [2]. Although they are capable of integrating traditional and digital media in communication, they tend to prefer email and face-to-face interactions [2]. With extensive clinical experience and a high level of confidence in their professional roles, Generation X NMs often emerge as motivational leaders. The values they emphasize, e.g., self-discipline and a strong work ethic, further strengthen these leadership qualities [3]. They are generally pragmatic and independent in character [11], valuing job security [12, 13], and social respect and support for traditions (altruism, existential and cognitive meanings, and self-realization), while not being driven by financial motives [13]. However, such a leadership style, rooted in experience and confidence, may also hinder adaptability and innovation [3].

Following Generation X, Generation Y (Millennials) is gradually assuming the role of NMs. Referred to as “digital early adopters,” they are a generation that grew up alongside the internet and digital media, and they tend to communicate through various digital platforms [2]. Compared with Generation X, Generation Y demonstrates greater openness to change, excels in embracing new ideas and driving organizational innovation, and is proficient in using technology while being motivated by personal growth, meaningful work, and social impact [3]. Millennial managers value collaboration; flexibility; and a transparent, relaxed, and balanced work environment. They are confident, reflective, and driven by feedback and achievement [14]. However, despite their confidence, owing to a lack of management experience [15], they may still fear failure when facing new or complex roles [16].

Generation Z, the youngest in today's workforce, are digital natives [2, 5] known for their quick responsiveness, rapid access to information, and active content seeking [5]. They prefer instant messaging, short videos, and multimedia content and are accustomed to real-time connection and interaction through mobile devices [14]. However, their frequent reliance on virtual communication [12] has led to a reduction in face-to-face interactions, which may hinder the development of interpersonal relationships [1, 5, 17]. Additionally, they tend to prefer working independently rather than collaboratively [14, 18] and often exhibit resistance to authority while prioritizing happiness in the workplace [19]. Compared with Generation X, Generation Z is no longer attracted to the traditional concept of a “fun workplace” [2]. Instead, they place greater value on flexible working hours, paid time off, and an attractive work environment [14]. Generation Z tends to pursue diverse work experiences and personal development. In contrast to Generation X, Generation Z is less likely to perceive the workplace as a space for long-term belonging or commitment. These differences in values partly explain Generation Z's higher turnover rates [6, 12].

Studies have shown that while 62% of newly graduated nurses enter clinical practice, 30% leave within 1 year and 57% within 2 years [20]. The World Health Organization [21] predicts a global shortage of approximately 4.8 million nurses and midwives by 2030. Nurse shortages and high turnover rates have significantly impacted the stability of patient care [20, 22], and NMs play a critical role in influencing nurse retention [23, 24]. In this context, understanding the characteristics of different generations and embracing their differences are essential for developing effective retention strategies [25]. Managers are also encouraged to leverage the commonalities and strengths of multigenerational teams to enhance collaboration and workplace cohesion [25]. For example, Generation Y shares communication styles similar to Generation X [1], while their values align more closely with Generation Z [1, 14, 18]. As such, Generation Y can serve as a bridge between Generations X and Z, helping NMs from Generation X better understand younger staff and supporting Generation Z in adapting to organizational culture. This type of intergenerational collaboration contributes to greater workplace stability and improved quality of care [1].

Generation Z is starting to influence the healthcare workforce, but its full impact remains unclear [11, 26]. What are Generation Z nurses seeking in the workplace? This is a question that NMs will need to answer in the coming years [26]. While some studies have examined Generation Z nurses' work values, communication, and job satisfaction [1, 2, 18, 27, 28], research on how Generation X NMs can effectively lead them remains limited. While existing literature has focused on the characteristics and needs of Generation Z, less has been given to actual challenges and experiences faced by Generation X NMs. Therefore, this study aimed to address the gap in the existing literature by exploring the experiences and growth processes of Generation X NMs in leading Generation Z nurses to provide practical insights for intergenerational workforce management.

## 2. Materials and Methods

### 2.1. Study Design

The epistemological foundation of this study is constructivism, where meaning and experience are created through social interactions [29]. We used inductive thematic analysis (TA) to identify and describe core themes across the interviews, deepening our understanding of participants' experiences and perspectives [30]. This study was reported in accordance with the Consolidated Criteria for Reporting Qualitative Research (COREQ) guidelines [31].

### 2.2. Participants

This study was conducted at a medical center in northern Taiwan with over 3100 beds. The nursing department's managerial structure comprises a director of nursing, deputy directors, supervisors, NMs, and assistant NMs. Among the 82 NMs, only two are male. A purposive sampling approach was used to recruit participants. The inclusion criteria focused on NMs from Generation X (born between 1965 and 1980) who had at least 1 year of management experience and were working in units with Generation Z nurses (born after 1996). NMs overseeing specialized units, e.g., intensive care units and operating rooms, were excluded owing to the unique nature of their work environments. Their personnel management practices may differ from those in general wards, which could lead to heterogeneity in the data and further complicate the integration and interpretation of NMs' experiences. On the basis of the nursing department's scheduling records, the first author initially excluded 26 NMs working in specialized units and 3 NMs who had a peer relationship with the first author within the same specialty area, from a total of 82 NMs. After applying these exclusion criteria, the remaining 53 NMs were identified as potential study participants. Subsequently, the first author sent email invitations to 53 NMs who met the preliminary criteria. Of those contacted, 9 were from the BB generation, 4 were from Generation Z, 6 had less than 1 year of managerial experience, and 7 did not have Generation Z nurses in their units; these were excluded on the basis of the study's inclusion criteria. Additionally, nine declined to participate owing to pandemic-related concerns, and seven did not respond. Ultimately, 11 NMs who met the criteria agreed to participate in the study.

### 2.3. Data Collection

The first author, a Ph.D. student with expertise in qualitative research and 7 years of experience as an NM, conducted interviews and collected data from November 2020 to February 2021. Prior to the interviews, participants were fully informed of the study's objectives and procedures, and written informed consent was obtained. Interviews were scheduled at the participants' convenience, each lasting between 35 and 50 min. These face-to-face interviews followed a semistructured format to ensure consistency in data collection, as outlined in Table 1. The first author audiorecorded all interviews and transcribed them verbatim within 1 week. Data collection and analysis were conducted concurrently until thematic saturation was reached, which was defined as the point at which no new themes emerged and all research questions had been satisfactorily addressed. To acknowledge participants' time and contributions, small gifts were offered as a token of appreciation.

### 2.4. Data Analysis

Data collection and analysis were conducted concurrently. The first author completed verbatim transcription of the interviews, and another researcher (Chin-Ying Huang) reviewed the transcripts for accuracy by comparing them with the original recordings. An inductive TA was performed following the six-phase process outlined previously [29, 30]. In the data familiarization phase, the first author and Hsiu-Chu Hsu repeatedly read the interview transcripts and documented their initial impressions through notes and memos to gain an in-depth understanding of the data. During the initial coding phase, they independently conducted open coding based on the research objectives, highlighting meaningful text segments and meaning units, and performed preliminary categorization to capture underlying concepts. In the theme-searching phase, they grouped similar codes into subthemes and overarching themes to reflect participants' experiences. In the reviewing phase, they jointly engaged in repeated discussions and comparisons of the initial themes, thoroughly examining the connections between themes and the original data to reach a consensus and construct the thematic framework. In the defining and naming themes phase, a peer review was conducted by co-authors Li-Hwa Lin and Shiow-Ching Shun. Shiow-Ching Shun, a nursing professor from another institution, provided a neutral third-party perspective that helped clarify theme meanings and confirmed the coherence of the thematic structure. Finally, the full research team finalized the themes through discussion. All interviews were conducted and transcribed in Chinese. For reporting purposes, the first author translated the codes, representative excerpts, and themes into English. Subsequently, a bilingual teacher performed back-translation into Chinese to ensure consistency with the original transcripts and accurate conveyance of meanings, thereby enhancing the credibility and confirmability of the study findings.

### 2.5. Trustworthiness

We adhered to the following four criteria established by Guba and Lincoln for assessing the rigor of this research: credibility, transferability, dependability, and confirmability [32]. Credibility was enhanced through member checking, whereby participants reviewed and validated the interpretations to confirm that their intended meanings were accurately represented. To enhance transferability, a detailed description of the research context was provided, including investigator and participant profiles, sampling methods, and data collection procedures. Dependability was supported through systematic documentation of the research design and procedures, with an audit trail maintained to record key analytic decisions. For confirmability, a qualitative research expert collaborated with the research team to review the transcripts and coding procedures, thereby enhancing analytical accuracy and reducing subjective bias and grounding the findings in the evidence provided by the data.

### 2.6. Ethical Considerations

The study was approved by the Institutional Review Board of the hospital (2019-07-007CC#1) and adhered to the ethical principles outlined in the Declaration of Helsinki. If participants felt distressed by any questions, the interview was stopped, and they were informed of their right to withdraw from the study, with assurances regarding the confidentiality of their information. To ensure confidentiality, all identifying information, including participants' names, ages, work units, years of service as NMs, and any revealing contextual details, was replaced with codes. These codes were consistently used throughout the data files, transcriptions, and analysis, with only the first author having access to the original data and the code key.

## 3. Results

### 3.1. Demographic Characteristics of Participants

In this study, 11 NMs from Generation X, aged between 47 and 55 years, were enrolled, all of whom were female, with 8 being married and 3 being single. Among the participants, 1 held a bachelor's degree, while 10 had master's degrees. Among the participants, six were in internal medicine, three in surgery, one in combined internal and external medicine, and one in pediatrics. Their experience in unit management ranged from 3.3 to 13 years (Table 2). Although there was consideration to invite male NMs to participate in the interviews, there were only two male NMs in the study hospital: one, who worked in the operating room, was excluded, and the other was excluded because he had less than 1 year of experience as an NM. Ultimately, only female NMs met the inclusion criteria and participated in this study.

### 3.2. Themes and Subthemes

The four themes, along with their associated subthemes derived from the participants' experiences and examples of coded phrases, are shown in Table 3.

#### 3.2.1. Comparative Experiences

In this section, participants reflected on their experiences as nurses and as NMs, drawing comparisons between their own professional journeys and those of Generation Z nurses. The two subthemes are as follows: (1) comparison with my own experience; (2) comparison of nurses across generations.

##### 3.2.1.1. Comparison With My Own Experience

In terms of work attitude, participants highlighted the substantial impact of parental upbringing and expectations on children's work attitudes. Parental values influence not only children's behavior but also their level of commitment to their careers, which is particularly evident in Generation Z nurses.

“Nowadays, most families have only one or two children. Parents of Generation Z are also still in the workforce. If a job is particularly demanding, parents of Generation Z would be willing to support their children's decision to leave. However, what our parents mainly focused on was to raise and support us until we graduate. After that, we are expected to be self-sufficient. Therefore, we can endure any hardship.” (Participant 3)

Participants would also compare their past positive or negative experiences. These comparisons assist them in making decisions when faced with similar situations by drawing on their own experiences as a reference. Positive experiences offer strategies and methods for problem-solving, while negative experiences serve as reminders for participants to exercise greater caution and avoid repeating past mistakes.

“I once encountered an NM who frequently showed favoritism toward a particular nurse, which left me feeling overlooked and frustrated. Generation Z staff members also often come to me with concerns about fairness regarding scheduling and case assignments. As a result, I strive to ensure that every nurse is treated equitably.” (Participant 10)

Some participants have Generation Z children, and their interactions with Generation Z family members have led them to exhibit greater experiential empathy.

“My child belongs to this generation and happens to be a nurse as well, so I often compare her with the new nurses I work with. They really do tend to focus more on themselves and place greater importance on their own rights. My child behaves this way too, so I am more able to understand and accept the behavior and attitudes of Gen Z nurses.” (Participant 1)

##### 3.2.1.2. Comparison of Nurses Across Generations

In the current clinical environment, nurses from Generations X, Y, and Z work together. Regarding Generation X colleagues, participants emphasized their stability and maturity; Generation Y exhibits strong work capabilities but tends to be less willing to take on managerial responsibilities; Generation Z generally pursues personal value, holds clear stances, and is less inclined to compromise. Participants indicated that they have a greater understanding of Generation X and Y nurses than Generation Z nurses.

“Generation X nurses possess certain typical characteristics; they tend to be more accommodating and non-confrontational and are generally more willing to follow instructions. Even when it requires working overtime, they are willing to cooperate.” (Participant 1)

“Some middle-generation (Generation Y) nurses are apprehensive about taking on management roles. They are not afraid of working; rather, they fear the potential conflicts that management entails as it often leads to offending others and making them unhappy.” (Participant 7)

“The difficulty in managing Generation Z staff lies in the absence of a one-size-fits-all approach. When a patient expresses a preference against a particular nurse's care, nurse A may agree, while nurse B might disagree, believing, “I haven't done anything wrong; why should I be replaced?” It is challenging to persuade everyone with a single argument.” (Participant 3)

#### 3.2.2. Dialectics of Value

This section explores the transformation process of the values held by Generation X NMs. Their values have long been shaped by traditional nursing ethics and a hierarchical workplace culture, with a particular emphasis on professionalism and discipline. However, with changes in the healthcare environment and the rise of Generation Z, their values have gradually begun to shift in response to external pressures and internal reflections. This transformation has not been easy; they have strived to move away from authoritative leadership styles toward more collaborative and empathetic approaches, a process characterized by tension, negotiation, and ongoing adjustment. The three subthemes are as follows: (1) conflict of values, (2) exploring the bottom line, and (3) preservation of face and image.

##### 3.2.2.1. Conflict of Values

Participants place great importance on nursing ethics and professional standards, which have guided their decision-making throughout their careers. In contrast, Generation Z tends to follow personal work principles that may not fully align with established norms. To maintain workplace order, participants emphasized the need for clear behavioral guidelines. One participant mentioned

“Sometimes when they approach you for communication, they have already predetermined the answer they want. If they do not receive a satisfactory response, they may become very upset. However, I believe that we must adhere to principles; we cannot simply comply with their demands. We must still operate according to established principles.” (Participant 6)

##### 3.2.2.2. Exploring the Bottom Line

Generation Z nurses occasionally make requests or exhibit behaviors that challenge the boundaries deemed acceptable by NMs. In navigating these situations, participants redefined their own limits of tolerance. This interactive process encouraged the development of more open communication channels and a more flexible, empathetic response to Generation Z's needs.

“When I advocate for policies or need to make changes due to policy requirements, the most common question they ask is, “Why? Why do we need to change?” At that point, you cannot simply say, “It's a directive from above” or “That's just how it is!” You must patiently explain to them why these changes are necessary and what their significance is, until they accept it.” (Participant 5)

##### 3.2.2.3. Preservation of Face and Image

Some participants indicated that they are not afraid of challenges from colleagues. However, when confronted with differing opinions, they tend to prepare in advance, anticipate possible scenarios, and respond cautiously to enhance their persuasiveness. This approach helps maintain a professional image and avoid loss of face, reflecting a shift in NMs from directive to consultative leadership, which in turn fosters team trust.

“Those who often criticize and enjoy expressing their opinions are, in fact, invested in the team. However, before communicating with them, I need to prepare for various scenarios and carefully consider how to present my views to avoid being dismissed. Being repeatedly rejected not only causes a loss of face but may also undermine my leadership effectiveness.” (Participant 7)

#### 3.2.3. Sense of Mission and Social Responsibility

In the current healthcare environment, the shortage of nurses is a critical issue. This situation has made participants profoundly aware of the heavy responsibilities and missions they bear, as well as their roles within this context. The four subthemes are as follows: (1) stay true to the initial intention, (2) awareness of leadership consciousness, (3) generational reconciliation and integration, and (4) efforts to retain Generation Z.

##### 3.2.3.1. Stay True to the Initial Intention

Participants describe themselves with metaphors such as “octopus” “having three heads and six arms,” indicating that they must meet the nursing department's standards and cultivate a positive work atmosphere. They embody flexibility, which requires them to be gentle at times and assertive at other times, taking on several challenging tasks simultaneously. However, the strength that sustains NMs is the combination of returning to simplicity and not forgetting their original intentions. This dual belief enables them to remain steadfast and composed in the face of complex personnel management challenges.

“Why do I continue to work so hard for so long? It's because the many wonderful experiences in patient care motivate me to stay in this field. When you have that motivation, your nursing career can be sustained for the long term.” (Participant 6)

“I believe we can only do our best. At the very least, as NMs of this generation, we still have certain responsibilities and missions regarding nursing.” (Participant 2)

##### 3.2.3.2. Awareness of Leadership Consciousness

Awareness of leadership consciousness refers to participants' re-evaluation of their roles and recognition that their behaviors and actions may significantly influence the retention of Generation Z, thereby fostering a heightened sense of responsibility. This awareness prompts NMs to contemplate questions such as “Who am I as a manager?” and “What should I do?” This not only enhances NMs' self-awareness but it also guides their leadership direction.

“I once read a book that stated a good leader must start with self-awareness … My feeling is that you can lose to others, but you must not lose to yourself. You must first understand your own shortcomings before you can identify areas for improvement, because you cannot be perfect; thus, you must also accept the imperfections of your colleagues.” (Participant 6)

Participants perceive “themselves” as management tools that influence their colleagues to engage in expected behaviors or avoid undesirable performances. One participant mentioned

“The way NMs behave will reflect on how their colleagues behave! Therefore, I am very mindful of my words and actions; I cannot allow my colleagues to adopt undesirable behaviors, such as complaining when faced with problems, as this will lead them to complain as well, which can negatively impact team morale.” (Participant 8)

Awareness of leadership consciousness encourages NMs to engage in continuous learning and growth. They actively seek learning resources to gain a deeper understanding of Generation Z's behavioral patterns and communication styles, thereby making their management approaches more refined and grounded.

“In addition to the management courses regularly offered by the hospital, I also seek out courses on management and personal growth. These courses involve extensive case discussions and experience sharing, which provide me with valuable learning and inspiration.” (Participant 3)

##### 3.2.3.3. Generational Reconciliation and Integration

In the face of work-related stress and generational differences, NMs had two options: to retire early or to remain in their roles and learn how to interact with Generation Z. Most participants chose the latter and began self-adjusting. They no longer relied solely on their own experiences for judgment, but instead sought to accept Generation Z for who they truly are, rather than viewing them through idealized or preconceived lenses. Once they let go of existing frameworks, many behaviors and perspectives of Generation Z that had previously been difficult to accept became easier to understand and embrace.

“I no longer view Generation Z through the lens of my own experiences; they are simply who they are. Once I started thinking this way, many of the things that used to confuse me became much easier to accept.” (Participant 6)

Participants began to realize that the only constant is change, and that embracing it is essential for moving forward in a workplace shaped by diverse values. In the process of generational integration, change is not a denial of past experiences, but a transformation of existing knowledge and expertise into new forms to meet the evolving demands of work and interpersonal interactions.

“Change doesn't mean I have to throw out everything I used to do; it's about adapting it so it remains meaningful and effective in a new context.” (Participant 10)

After transcending generational divides, participants found that they could interact with Generation Z in a calmer and more composed manner and gradually began to recognize the advantages brought about by change.

“In recent years, our unit has undergone many changes, such as modifications to ward rules and scheduling principles. These different perspectives have often been put forward by Generation Z nurses, and sometimes, changes can be beneficial.” (Participant 2)

Participants emphasize that interacting with Generation Z requires not only expressing concern but also building rapport. The concern is often one-sided, with one party showing care for the other, whereas building rapport involves mutual interaction. Therefore, they aim to establish emotional connections with Generation Z through words and actions, hoping that such interactions will foster mutual recognition, trust, and respect.

“Management comes with many responsibilities and demands; we are people who constantly raise expectations, while nurses are those who are exhausted from meeting our demands. If we want to connect with them, we need to set aside our title, integrate into their communication style, and listen to their thoughts.” (Participant 3)

##### 3.2.3.4. Efforts to Retain Generation Z

Generation Z learns quickly about new things but still lacks depth and nuanced understanding. Therefore, a gap remains between “learning” and actually “doing,” as well as “doing it correctly.” They are less likely to proactively ask questions, which necessitates that NMs take the initiative to provide the necessary support and assistance to help them better master patient care skills.

“Some new nurses will say there are no problems when caring for patients, but my observations tell a different story. Therefore, I take the initiative to guide them through the process, working alongside them and encouraging them to practice several times until they truly master the necessary skills.” (Participant 6)

Additionally, some participants noted that they have exerted their utmost efforts to retain new staff, using various potential methods and strategies. What is then required is simply patience as they await the outcomes. A strong psychological resilience is also an essential prerequisite for the growth of NMs.

“For some new nurses who learn slowly, those around them may hint at their potential for failure. Yet, if you do not give up on them, they may suddenly have a breakthrough, not only succeeding in their roles but also performing exceptionally well. You feel grateful that your persistence and patience were justified.” (Participant 1)

Ultimately, participants believe they have the responsibility to guide new colleagues in experiencing and practicing the true, good, and beautiful aspects of nursing, helping them create greater meaning and value in their work.

“Nursing cannot simply be about completing daily routine tasks; it must involve guiding them to understand the life and value of nursing. I believe these are the motivations that support them in moving forward; otherwise, they can easily be overwhelmed by the complexities of clinical duties.” (Participant 10)

#### 3.2.4. Support System

In the past, participants were accustomed to a traditional authoritarian leadership model, where they followed directives and issued commands. However, this management model is no longer applicable. Therefore, participants expressed their needs within the new management model, identifying two subthemes as follows: (1) emotional support and (2) substantive support.

##### 3.2.4.1. Emotional Support

Participants expressed a profound awareness of the negative impact that staff shortages have on patient safety and quality of care. With limited human resources, they need to not only retain new nurses but also reassure the existing nursing staff. They emphasized the importance of emotional support for NMs under multiple pressures as it helps them maintain calmness and objectivity in high-stress situations.

“Colleagues have their emotions, patients have their emotions, and NMs have emotions as well. When my emotions are soothed, I am able to address the issues of both my colleagues and the patients.” (Participant 10)

However, when their needs are not met with understanding or support, it can leave NMs feeling isolated and helpless, ultimately affecting their motivation and performance at work.

“We need a superior who can support all our thoughts and decisions. Whenever you try to promote something, if your superior's level of agreement differs from yours, it increases the difficulty of persuading your colleagues, and their trust in you will also decline.” (Participant 9)

##### 3.2.4.2. Substantive Support

Participants emphasized that retaining new nurses is not only the responsibility of the nursing management but also a shared responsibility of the entire healthcare team. Through collaboration and mutual support, a work environment that is conducive to the adaptation and growth of new nurses can be created, which is crucial for improving nurse retention. One participant mentioned

“The cardiac surgery unit is a highly specialized department. If new nurses lack sufficient expertise, they feel significant pressure during physician rounds. To address this, we invite doctors or specialized nurses to teach our staff, and they are willing to mentor new nurses. This not only facilitates the learning of new nurses but also alleviates my own stress.” (Participant 3)

## 4. Discussion

This study employed qualitative research methods to explore the adaptation process and growth experiences of Generation X NMs leading Generation Z nurses, identifying four main themes: comparative experiences, dialectics of value, sense of mission and social responsibility, and support system. These themes highlight the challenges of intergenerational leadership and the strategies Generation X NMs adopt in response. While previous studies have acknowledged the difficulties posed by generational diversity in nursing leadership [14, 25], this study is the first to focus on the proactive efforts, repeated adaptations, and growth processes of Generation X NMs in their interactions with Generation Z nurses. Through these experiences, we not only deepened our understanding of intergenerational interactions but also contributed new evidence-based insights to the fields of nursing leadership and generational management. Furthermore, we provided future perspectives for healthcare systems and senior nursing management on strategies to retain Generation Z nurses.

### 4.1. Comparative Experiences

Our findings reveal that “experience comparison” is the most common thinking pattern among Generation X NMs when assessing Generation Z nurses. This reflective approach is also evident in the leadership practices of Generation Z NMs, who also tend to incorporate their life experiences and early career trajectories into their management strategies [14]. Although generational backgrounds differ, culture and knowledge are passed down through generations [1], and the NMs from Generations X and Y still exhibit several commonalities in their leadership styles. Similarly, Generations Y and Z share many commonalities, particularly in terms of work–life balance, the pursuit of a sense of achievement, supportive work environments, and the use of frequent feedback to foster professional development [1, 14, 18]. Based on these commonalities, Generation X NMs can indirectly influence the work attitudes and behavioral performance of Generation Z by closely collaborating with Generation Y, which serves as the bridging generation, thereby promoting intergenerational understanding and collaboration [1].

### 4.2. Dialectics of Value

In this study, value conflicts were identified as a substantial challenge faced by Generation X NMs. The conflict primarily arises from their insufficient understanding of the work attitudes and philosophies of Generation Z. They indicated that they often find it difficult to understand the thoughts and working styles of Generation Z. Förster et al. [33] pointed out that hospital leaders are influenced by the values, ideas, and work philosophies of different generations, which can easily lead to internal tensions. Conflicts can lead to stress, affecting patient safety and quality of care, reducing nurses' engagement in their work [1], and negatively impacting the management performance of NMs [34, 35]. However, NMs who have Generation Z children at home, being familiar with their communication styles and thought patterns, experience fewer conflicts. The key to overcoming value conflicts lies in effective communication [1]. When communicating with Generation Z, it is important to use language and expressions they can understand [36]. For example, using communication tools such as internal networks and instant messaging can help provide information in a full, authentic, and concise manner, which will facilitate effective communication [10, 11]. Regular feedback is a crucial part of maintaining good communication [14]. Providing feedback that shows care and support for their experiences [10] helps to ensure that both parties understand each other's expectations and needs, thereby fostering a positive work environment and collaborative atmosphere.

During the process of dialectical values, participants further pointed out the dual significance of maintaining image and face. First, they placed a high value on professional image, viewing it as the cornerstone for NMs to establish trust and confidence in the healthcare setting. Confidence enables individuals to make correct decisions when facing external challenges and enhances leadership effectiveness [33]. Second, “face” is an extremely important and influential social concept in Taiwanese culture, encompassing personal dignity, social status, and the perceptions of others. It is deeply embedded within interpersonal relationships and social interactions [37]. Therefore, losing face can lead to an individual being demeaned or disrespected in social and professional settings, often triggering feelings of shame or embarrassment [38]. To safeguard face and uphold their professional image, NMs frequently take proactive steps to foster constructive interactions with Generation Z staff, thereby minimizing the potential for conflict. Respect is a generational commonality [2]. Just as NMs have the responsibility to care for the dignity of patients and their staff [39], they also need to be respected by team members, whether superiors, medical teams, colleagues, or even patients. Only with respect can NMs effectively fulfill their leadership roles, promote teamwork, and enhance the quality of healthcare services.

### 4.3. Sense of Mission and Social Responsibility

A noteworthy finding of this study is that, driven by a sense of mission and social responsibility, NMs have developed a profound understanding of cross-generational reconciliation and integration. Participants mentioned how they leverage these driving forces to overcome the impacts of generational differences, alleviate personal emotional responses, and maintain a positive attitude even under pressure. They enhance collaboration by learning about Generation Z's culture, dialog, and cooperation.

The subtheme of “staying true to one's initial intentions” is viewed as a key factor influencing whether NMs can continue to remain in their positions. “Initial intention” can be understood as the initial inner motivation a person has when engaging in a particular activity or choosing a profession. This motivation can inspire oneself and others, helping them develop effective coping strategies. Participants indicated that serving patients is the core motivation for their nursing work. This motivation constantly reminds them of the value, joy, and meaning of being a nurse, helping them maintain enthusiasm and determination during difficult times. This finding aligns with previous research indicating that NMs first identify themselves as nurses, with the primary purpose of being a nurse being to help patients [35]. Patients are the source of their happiness and meaning; even if they are no longer directly involved in daily patient care, patients remain the central focus for NMs [40]. However, in today's increasingly complex healthcare setting, to maintain their initial intent, healthcare institutions and policymakers should adopt necessary strategies, such as establishing a safe communication platform where NMs can freely share their experiences. Sharing experiences can enhance inner strength [39], and peer learning through experience sharing not only increases NMs' job satisfaction but also contributes to improving their willingness to retain.

### 4.4. Support System

Participants pointed out that when NMs receive support from all levels of the organization, including hospital administration, interdepartmental collaboration, and within-team support, it not only facilitates the adaptation and professional growth of newly recruited nurses but also significantly alleviates the stress NMs face in their work. Qualitative research focusing on Generation Y NMs also emphasized the importance of support from director [16]. Support can be categorized into several dimensions including economic and technical assistance, material support, emotional care, social interaction capacity, and peer support from individuals with similar experiences [41]. Among these, support from superior managers is considered a key factor in predicting the well-being of NMs [39]. Feedback, active listening, and trust from superior managers can help NMs effectively fulfill their responsibilities [22].

In addition, organizational support plays an essential role. Frangieh et al. [9] reported that NMs generally perceived the level of organizational concern for their job satisfaction as moderate. Organizational support positively influences interpersonal relationships, communication quality, job performance, and innovative behaviors [41] and can effectively reduce feelings of loneliness [9]. When managers perceive strong organizational support, they are more likely to demonstrate high levels of organizational commitment and adopt supportive leadership behaviors [42], thereby strengthening their leadership effectiveness and team cohesion.

Nurse shortages are a serious issue, and high turnover rates further exacerbate this dilemma, putting greater pressure on the healthcare system, affecting the quality of patient care, and increasing the burden on existing staff [43]. To help NMs successfully retain newly recruited nurses, healthcare institutions must provide them with adequate resources and support, including mentorship, professional training, emotional support, and improvements to the work environment [33].

## 5. Limitations

Some limitations of this study need to be noted. Data for this study were collected during the early stages of the coronavirus pandemic, prior to its widespread outbreak; therefore, NMs' experiences during the pandemic peak or postpandemic period are not included. The pandemic has caused substantial disruptions to healthcare delivery and management worldwide [44], e.g., the shift from in-person teaching to online learning. While Generation Z has grown up in a digital age, the mandated social distancing and prolonged engagement in digital learning have still posed psychological challenges, including feelings of loneliness, anxiety, and stress [45]. These concerns may further influence management practices and intergenerational interactions. Additionally, further research is needed to explore how the pandemic has reshaped intergenerational interactions and management strategies, particularly how NMs address the psychological needs of Generation Z nurses in the postpandemic era. Due to the predominance of female NMs in the participating institutions, male participants could not be included during the data collection period. Consequently, all participants in this study were female, which may have led to a sex-biased sample. Therefore, the findings primarily reflect the experiences of female NMs in leading Generation Z nurses, and this sex homogeneity may limit the generalizability of the results. As gender beliefs and stereotypes may influence leadership styles and behaviors [46], future research should include male NMs to provide a more comprehensive understanding of generational leadership experiences and to address this study's limitations. The first author had a professional background as a NM and was employed at the hospital where the study was conducted during the research period. This background facilitated a deeper understanding of the interview content but may have also influenced the analysis results. Therefore, participant response bias could not be completely ruled out. To minimize these potential influences, the first author consistently applied the bracketing strategy throughout the data collection and analysis process, consciously setting aside personal preconceptions and professional assumptions. This strategy was implemented through diary writing, memoing, self-reflection, and discussions with peers to clarify the researcher's own standpoint and preconceived views [47]. These practices supported more thorough analysis and reflection, thereby revealing deeper meanings underlying the studied phenomena.

## 6. Conclusions

This study reveals that Generation X NMs underwent a profound journey of self-awareness, encompassing reflection, internal dialog, and dialectical of values. They gradually recognized that their previous management mindset could no longer meet the expectations and needs of the new generation of nurse, prompting them to initiate change. This transformation is not a rejection of existing values, but rather an effort to seek balance between traditional and emerging perspectives, thereby fostering greater understanding and integration across generations. In the face of increasingly severe nursing workforce shortages, Generation X NMs demonstrated a strong sense of mission and responsibility, actively engaging in the retention and development of Generation Z nurses. Despite bearing the dual pressures of generational differences and work-related stress in their leadership roles, most maintained a positive attitude and remained committed to their positions. Their “initial intention” and support from superiors served as essential sources of strength that sustained their efforts. Moreover, through continuous learning and practice, NMs strengthened their management capabilities to adapt to the ever-changing clinical and healthcare environment, aiming to develop a more flexible and inclusive leadership style. This journey not only reflects personal growth and transformation but also highlights the potential for generational inclusion within organizations. The experiences of Generation X NMs offer valuable practical insights and provide direction for workplaces where multiple generations coexist, contributing to the development of a more resilient and collaborative leadership culture.

## 7. Implications for Nursing Management

This research makes several contributions to the medical field. First, it provides evidence for healthcare policymakers that, when developing nurse retention policies, they must also consider the needs of NMs and provide them with more comprehensive management training programs and emotional support to enhance their key management skills and qualities. Second, the results of this study can be used as a reference for NMs with similar experiences or newly appointed NMs, which may help them retain passionate young nurses and develop workplaces that meet the needs of Generation Z. Finally, considering that Generation Z will soon join the medical team in large numbers, future research should focus on the experiences of this generation of nurses and compare them with the experiences of managers to understand the differences in their perspectives and more effectively promote intergenerational cooperation and integration, thereby improving nurses' job satisfaction and retention rates and providing better quality care for patients.

## Figures and Tables

**Table 1:** Interview guide.

(1) Does your unit have any nurses born after 1996 from Generation Z?
(2) What is your perspective on Generation Z nurses?
(3) What do you consider the most challenging aspects of leading Generation Z nurses?
(4) What do you believe is the most important when leading Generation Z nurses?
(5) Based on your experience, what characteristic should a nurse manager possess?
(6) Please describe what a nurse manager is using your own adjectives?
(7) How do you perceive the role of a nurse manager?
(8) Finally, could you share some memorable experiences in leading new-generation nurses?

**Table 2:** Demographic characteristics of the participants (*N* = 11).

No.	Age (years)	Sex	Marital status	Degree	Unit	Years in NM
1	51	F	Married	MSN	Med	11
2	51	F	Married	BSN	Ped	13
3	49	F	Married	MSN	Surg	10
4	54	F	Unmarried	MSN	Med	14
5	51	F	Married	MSN	Surg	6.5
6	47	F	Married	MSN	Med	5
7	51	F	Unmarried	MSN	Med	1.5
8	52	F	Married	MSN	Med Surg	7
9	51	F	Married	MSN	Med	3.3
10	55	F	Unmarried	MSN	Surg	12
11	46	F	Married	MSN	Med	3.4

Abbreviations: Med = Medical, NM = Nurse manager, No. = Number, Ped = Pediatrics, Surg = Surgical.

**Table 3:** Themes, subthemes, and example phrases.

Theme	Subtheme	Example of CODED phrases
(I) Comparative experiences	(1) Comparison with my own experience	Parents' parenting styles and expectations can influence their children's work attitudes.
		Generation Z is more willing to express their thoughts, whereas our generation tends to be more restrained and conservative.
		I lead my staff the way my NM leads me.
		My previous experiences influenced my view of generation Z personnel.
	(2) Comparison of nurses across generations	Compared with nurses from generations Y and Z, nurses from generation X have higher maturity and job stability.
		Generation Y nurses tend to prefer self-management and do not like to manage others.
		Generation Z has its own unique thinking and viewpoints, not easily persuaded.
		Generation Z interacts more with generation Y than with generation X, and the conflicts between them are fewer.

(II) Dialectics of value	Conflict of values	They will not listen to you just because you are the NM.
		They determine their own ways of communication and behavior, regardless of what you say.
		They hold a questioning attitude toward the existing order and authority.
		To establish clear and explicit behavioral rules.
	Exploring the bottom line	You may feel like they challenge you repeatedly.
		When you say one thing, they might respond you with ten.
		I feel like they are testing the limits of my tolerance.
		I am also exploring my own boundaries.
		I often hover between persistence and compromise.
	Preservation of face and image	It is embarrassing to be shot down when communicating with new staff.
		If you keep getting shot down by staff, leading them becomes difficult.

(III) Sense of mission and social responsibility	Stay true to the initial intention	The ward is my second home.
		Engaging with patients often leads to unforeseen rewards.
		Nursing requires inheritance.
		You have a responsibility to fulfill initial commitments.
	Awareness of leadership consciousness	I ask myself if I have the traits that make staff want to follow?
		If you do not possess leadership abilities, why would staff follow you?
		My responsibility is to impart the correct nursing values to my staff.
		Enhancing my professional and management skills.
	Generational reconciliation and Integration	I no longer view Generation Z through the lens of my own experiences.
		The only constant is change.
		Change is about transforming, not denying, the past.
		Recognize the advantages brought about by change.
		We need to set aside our title and integrate into their communication style.
	Efforts to retain Generation Z	Provide hand-over-hand, step-by-step instruction when they do not understand.
		If they stay, I will give them more time to learn.
		Patiently wait for them to grow.
		You need to show passion and motivation at work to influence your staff.

(IV) Support system	Emotional support	I need to be understood, empathized with, and supported.
		I hope my superior will not negate my approach to training new staff in front of others.
		The trust from my superior is a great motivation for me.
	Substantive support	Support from the healthcare team is also a key factor influencing the retention of Generation Z.
		I appreciate that our doctors and specialist nurses are willing to teach new staff.
		I hope my superior can help me deal with the tricky personnel issues.

## Data Availability

The data from this study are accessible from the corresponding author upon reasonable request.
